# Validation of the Wijma Delivery Expectancy/Experience Questionnaire (Version B) Among Greek Postpartum Women

**DOI:** 10.3390/healthcare13080896

**Published:** 2025-04-14

**Authors:** Pinelopi Varela, Ioannis Zervas, Christina Nanou, Victoria Vivilaki, Aikaterini Lykeridou, Anna Deltsidou

**Affiliations:** 1 General Hospital of Athens “Alexandra”, 11528 Athens, Greece; 2 Department of Midwifery, University of West Attica, 12243 Athens, Greece; nanouxv@uniwa.gr (C.N.); vvivilaki@uniwa.gr (V.V.); klyker@uniwa.gr (A.L.); adeltsidou@uniwa.gr (A.D.); 3 First Department of Psychiatry, School of Medicine, National and Kapodistrian University of Athens, Eginition Hospital, 11528 Athens, Greece; izervas@med.uoa.gr

**Keywords:** fear of childbirth, postpartum, Wijma Delivery Expectancy/experience questionnaire, psychometric properties, validity, reliability, exploratory factor analysis, Greece

## Abstract

**Background/Objectives**: Postnatal fear of childbirth (FOC) has a significant impact on women’s mental health following childbirth. A widely employed tool for evaluating postnatal FOC is the Wijma Delivery Expectancy/Experience Questionnaire version B (W-DEQ-B). This study aimed to validate the Greek version of the W-DEQ-B (GrW-DEQ-B) and confirm its reliability among Greek postpartum women having a low-risk pregnancy. **Methods**: At four weeks postpartum, 200 mothers after a low-risk pregnancy completed questionnaires, which included the GrW-DEQ-B and the Edinburgh Postnatal Depression Scale (EPDS). **Results**: The majority of participants had a vaginal delivery (80%), 52.0% of the sample were primigravida, and the mean gestational age at delivery was 38.8 weeks (SD = 0.8). The exploratory factor analysis yielded six factors (“Lack of self-efficacy”, “Lack of positive anticipation”, “Lack of feeling lonely”, “Concerns about delivery and losing control”, “Calmness”, and “Concern for the child”) of 33 items from the W-DEQ-B. The instrument’s multidimensionality was verified by the confirmatory factor analysis (RMSEA = 0.07; CFI = 0.90; TLI = 0.88). All Cronbach’s alphas were over 0.7, indicating acceptable reliability of the factors. Almost all factors of the GrW-DEQ-B were significantly correlated with each other (*p* < 0.001), demonstrating the convergent validity of the tool. Significant correlations were found between almost all dimensions of the GrW-DEQ-B and the EPDS (*p* < 0.001), indicating the divergent validity of the tool. **Conclusions**: This study provides evidence that the Greek version of the W-DEQ-B proved to be a reliable and valid measure of FOC among Greek postpartum women.

## 1. Introduction

One psychological construct that falls under the general heading of anxiety during the perinatal period is the fear of childbirth (FOC) [1]. FOC may develop during the perinatal period with the manifestation of symptoms of worry, anxiety, or even extreme fear [2,3]. Although it is normal for perinatal women to feel some level of FOC [4], the fact that FOC exists on a spectrum from low to severe [5,6] does not reassure its appearance because some women’s FOC goes beyond simple anxiety [7]. The severe fear of pregnancy or giving birth with which these women struggle is referred to as “tocophobia” or “tokophobia”, and it is typified by extreme anxiety and a complete avoidance of thoughts and behaviors that go beyond what is deemed normal [7,8]. The theories behind FOC are the same as those regulating anxiety in general, according to the literature on the subject. There are three essential factors to the origin of anxiety disorders: a generalized biologic vulnerability that is inherited, a generalized psychological vulnerability, and a specific psychological vulnerability. All three of these factors are present in an individual who develops an anxiety disorder [9]. In accordance with research findings, the following key components underpin the FOC construct: fear of not knowing and not being able to plan for the unpredictable, fear of harm or stress to the baby, fear of inability to cope with pain, fear of harm to self in labor and postnatally, fear of being ‘done to’, fear of not having a voice in decision making, fear of being abandoned/alone, the body’s ability to give birth, fear of internal loss of control, and being terrified of birth and not knowing why [10]. According to literature data, the prevalence of FOC is rising [11,12], yet it is not given the proper recognition or priority as a health condition [6]. Based on the research data, 20% of women report having significant levels of fear [11], while FOC rates range from 6.3% to 14.8% among countries [13]. The global prevalence of severe FOC was 16%, up from 14% previously, according to recent research data [11,12].

In addition to determining the prevalence of FOC, the research activity that has been observed has also given attention to the consequences of FOC, which are not restricted to the antenatal period but also extend into the postpartum phase and beyond. In a broader sense, the impacts described in the literature include fear that intensifies to the point that the affected women are unable to carry out their daily responsibilities [14,15], the prolonged period of labor [16,17], the increased risk of cesarean section (CS) [18,19], and the negative birth experience [2]. Moreover, FOC can affect the childbirth decisions of women in the future [20] and can be the cause of women’s avoidance of circumstances and stimuli associated with future childbirth [21]. This avoidance and an effort to manage this severe or excessive fear may result in a request for a CS [22]. Additionally, it has been found that women who experienced prenatal FOC may experience FOC even one year after giving birth [23]. Also, women who experienced FOC in a previous pregnancy are more likely to experience FOC in the next pregnancy, thus causing a cycle of anxiety and depression symptomatology [24]. Anxiety or posttraumatic stress disorder symptoms during the postpartum period have been linked to women with postnatal FOC [25]. Furthermore, it has been observed that women who had the greatest levels of FOC throughout pregnancy also had the highest levels of FOC during and following delivery [26,27].

Given all mentioned so far, it is crucial to address FOC following childbirth. In view of this, it has previously been proposed that future researchers work to create suitable interventions meant to detect pregnant women who are at risk of FOC [11]. Additionally, it has been proposed that maternity care providers use validated tools to regularly test for FOC. In this manner, women who screen positive might receive early care and support [9]. Therefore, the establishment of the most valid and reliable screening tools or approaches has been mentioned as an important area of research [6]. The scientific community’s activities regarding the use of several psychometric tools related to FOC [13] suggest that this fear that affects women during the perinatal period has received attention. The most employed psychometric tool for the measurement of FOC is the Wijma Delivery Expectancy/Experience Questionnaire (W-DEQ) [11,13] and has drawn the focus of researchers given that it has been translated into at least 17 different languages [28]. Wijma et al. (1998) developed the W-DEQ to measure FOC properly since FOC is a psychological domain of its own. The W-DEQ includes versions A and B (W-DEQ-A and W-DEQ-B), which are designed for antenatal and postnatal use, respectively [29].

Thus far, our research team has validated the Greek version of W-DEQ-A (GrW-DEQ-A) [30]. In order to fill the gap regarding the assessment of FOC after childbirth among postpartum Greek women, the present study was carried out, which aimed at the evaluation of the psychometric properties of the Greek version of the W-DEQ version B. Midwifery care professionals in Greece will, thus, have the opportunity to measure FOC in a valid and comprehensive manner throughout the perinatal period.

## 2. Materials and Methods

### 2.1. Phase of Translation and Pilot Testing for Version B of the Wijma Delivery Expectancy/Experience Questionnaire (W-DEQ-B)

The translation process, which consisted of four stages—forward translation, synthesis of the translations, back translation, Expert Committee, and submission of documentation to the developer—began after the scale’s creator (Professor Klaas Wijma) provided approval to it [29]. The W-DEQ-B pilot test was conducted by asking the same sample group of thirty postpartum women to complete the questionnaire at different times. W-DEQ version B’s test–retest reliability (intraclass correlation coefficients, ICC) varied between 0.92 and 1.00, and Cronbach’s a reliability coefficient was 0.94. All the data from the pilot study have already been published [31]. The Greek version of the W-DEQ version B (GrW-DEQ-B, Table S1) emerged following the results of the pilot study.

### 2.2. Study Participants

Postpartum women who had given birth during the previous month were the study’s sample. A few criteria were used for inclusion: postpartum women who were adults with a sufficient understanding of Greek and had a low-risk pregnancy. The exclusion criteria were as follows: postpartum women who had a high-risk or multiple pregnancy, a severe chronic disease, a psychiatric illness, or were under psychiatric medication.

### 2.3. Study Procedure

This study was carried out from July 2020 to December 2021 at a public maternity hospital in Athens. Participants were invited to participate in the study by the principal researcher during their regular prenatal visits. The final sample consisted of two hundred of the two hundred and forty women who were initially invited to participate in this study. Before taking part in the study, each participant signed informed consent. The participants were instructed to fill out a data questionnaire (demographic and mental health data, obstetric history, and details about the recent delivery and postpartum period) and two psychometric instruments.

### 2.4. Measures

#### 2.4.1. Wijma Delivery Expectancy/Experience Questionnaire Version B (W-DEQ-B)

The W-DEQ-B is a self-assessment tool that evaluates the experience of childbirth to measure FOC following labor. All postpartum women may fill it out, regardless of whether they are primiparous or not. A six-point Likert scale, ranging from “not at all” to “extremely”, is used to score answers to the thirty-three items in the questionnaire. A total score, with zero being the lowest and 165 being the highest, is calculated by adding the scores of each of the items. It is necessary to reverse the scores for items 2, 3, 6, 7, 8, 11, 12, 15, 19, 20, 24, 25, 27, and 31. The higher the score is, the greater the FOC is demonstrated. The original version of W-DEQ-B appeared to have high reliability since the values of Cronbach’s alpha two hours after delivery (α = 0.93) and five weeks after delivery (α = 0.94) were both quite satisfactory, in addition to the values of split-half reliability (2 h after delivery: r = 0.95, 5 weeks after delivery: r = 0.96) [29]. The multifactorial structure of W-DEQ-B has been confirmed by factor analysis conducted by subsequent studies [32,33,34,35,36,37].

#### 2.4.2. Edinburgh Postpartum Depression Scale (EPDS)

The EPDS is a self-report instrument for the assessment of depressive symptomatology. Each of the four potential answers is ranked according to the severity of the ten items on the tool, which describe symptoms of depression. The sum of the responses’ scores is calculated once they are rated from 0 to 3 [38]. The Greek version scale’s internal consistency reliability is characterized by a satisfactory Cronbach’s alpha (α = 0.9) [39].

### 2.5. Statistical Analysis

To describe the qualitative variables, both the relative (%) and absolute (N) frequencies were used. The quantitative variables were described by the use of mean values (mean), standard deviations (SDs), medians (median), and interquartile ranges (IQRs). Using the maximum likelihood estimation method, confirmatory factor analysis (CFA) was performed to assess how well the W-DEQ-B one-factor model fit the data. As goodness-of-fit indices, the comparative fit index (CFI), the Tucker–Lewis index (TLI), and the root mean square error of approximation (RMSEA) were used. These indicators were considered acceptable when CFI ≥ 0.90, TLI ≥ 0.90, and RMSEA ≤ 0.05. To assess the construct validity of W-DEQ-B, an exploratory factor analysis (EFA) was conducted. The Kaiser–Meyer–Olkin (KMO) method with >0.6 considered acceptable and a significant Bartlett’s test of sphericity were used for the confirmation of the adequacy of the data. Principal component analysis (PCA) and varimax rotation were employed to identify factors and enhance the solution’s interpretability. An evaluation of the scree plot and an eigenvalue greater than one (>1) determined the number of factors that were retained. To determine if an item sufficiently represented its factor, a factor loading of ≥0.40 was applied [40,41,42,43]. The Cronbach’s alpha coefficient was calculated to assess the reliability of internal consistency. Reliability values of 0.70 or higher were considered appropriate [44]. Using Spearman’s correlation coefficient (r), the performances of the convergent and divergent validity as well as correlations between the GrW-DEQ-A and the GrW-DEQ-B were evaluated. The GrW-DEQ-B factors’ intercorrelations were used to examine convergent validity, while the EPDS was used to evaluate the scale’s divergent validity. The threshold for statistical significance was *p* < 0.05, and analyses were conducted using SPSS (version 26.0) and STATA (version 13.0).

## 3. Results

### 3.1. Characteristics of the Sample

This study’s sample consisted of 200 postpartum women, whose average age was 34.3 years (SD = 4.2). The vast majority of the sample was Greek (96.0%) and resided permanently in Athens (90.5%). A great proportion of the sample were employed (79%), were married/living with their partner (99.5%), and had a university degree (64%). One hundred eighty-seven women reported that their supportive environment was at a satisfactory level (93.5%). The majority of participants (87.5%) who had previously given birth had a vaginal delivery, and 53.2% of them described their previous childbirth experience as mainly positive. Primigravida were 52.0% of the sample, and the present pregnancy for 63% of the participants was planned. The mean gestational age at delivery was 38.8 weeks (SD = 0.8), most of the sample had a vaginal delivery (80%), with 53.5% of participants characterizing their childbirth experience as mainly positive, and 98% of newborns were full-term. The characteristics of the sample are presented in Table 1.

### 3.2. Confirmatory and Exploratory Factor Analysis

To investigate factorial validity, CFA was conducted for the unidimensional version of the W-DEQ-B, as proposed by its creators. The CFA results revealed a very poor model fit for the GrW-DEQ-B (CFI = 0.65; TLI = 0.63; RMSEA = 0.09). Therefore, to examine the internal structure of GrW-DEQ-B, EFA was conducted. A KMO of 0.89 and a significant Bartlett’s test, *p* < 0.001, confirmed the sample adequacy. EFA with varimax rotation produced six factors that explained 61.1% of the variance and were similar to those of the GrW-DEQ-A [30]. Factors «Lack of feeling lonely» and «Lack of self-efficacy» each consisted of 10 items. Factors «Lack of positive anticipation» and «Calmness» each consisted of four items. Factor «Concerns about delivery and losing control» consisted of three items and factor «Concern for the child» consisted of two items. Table 2 displays both their loadings and the variance explained by each factor. CFA was performed on the new six-factor solution of the GrW-DEQ-Β, as revealed by the EFA. CFA revealed an acceptable model fit for the GrW-DEQ-B (RMSEA = 0.07; CFI = 0.90; TLI = 0.88). Participants’ scores on the six dimensions of the GrW-DEQ-B appear in Table 3.

### 3.3. Internal Consistency of the GrW-DEQ-B

Cronbach’s α reliability coefficients were above 0.7 for all dimensions, indicating acceptable reliability. No items were eliminated since doing so would not increase the coefficients. Additionally, each item’s correlation coefficient with the overall score for every factor is considered acceptable (>0.3). Table 4 displays the item–total correlations and Cronbach’s α for each factor of GrW-DEQ-B.

### 3.4. Convergent and Divergent Validity of the GrW-DEQ-B

Almost all factors of the GrW-DEQ-B were significantly correlated with each other, demonstrating the convergent validity of the tool. The exception was the factor «Concern for the child», which was not significantly correlated with the factors «Calmness» and «Concerns about delivery and losing control». Table 5 presents the Spearman correlation coefficients between the six dimensions of the GrW-DEQ-B. The results of the assessment of the divergent validity of the GrW-DEQ-B dimensions with the EPDS are presented in detail in Table 6. Significant correlations were found between almost all dimensions of the GrW-DEQ-B and the EPDS. An exception was the dimension «Concerns about delivery and losing control», which was not found to be significantly correlated with the EPDS. The level of correlations found was low or very low.

### 3.5. Correlation Coefficients Between the GrW-DEQ-A and the GrW-DEQ-B

Significant correlations were also found between almost all factors of the two versions of the GrW-DEQ. The exceptions were the dimension «Lack of positive anticipation» of the GrW-DEQ-A, which was not significantly correlated with the dimensions «Calmness» and «Concern for the child» of the GrW-DEQ-B; the dimension «Calmness» of the GrW-DEQ-A, which was not significantly correlated with the dimensions «Concerns about delivery and losing control» and «Concern for the child» of the GrW-DEQ-B; and the dimension «Concern for the child» of the GrW-DEQ-A, which was not significantly correlated with the dimensions «Lack of self-efficacy», «Lack of positive anticipation», and «Concerns about delivery and losing control» of the GrW-DEQ-B. Table 7 presents the Spearman correlation coefficients between the dimensions of the two versions of the GrW-DEQ.

## 4. Discussion

The assessment of the psychometric characteristics of the Greek version of W-DEQ-B in postpartum women was the objective of the present study. In addition to factor analysis, internal consistency and convergent and divergent validity were examined. The main results indicated that the GrW-DEQ-B has a multidimensional structure, an acceptable internal consistency, and also significant correlations regarding convergent and divergent validity.

The GrW-DEQ-B comprises 33 items and has a six-factor structure («Lack of feeling lonely», «Lack of self-efficacy», «Lack of positive anticipation», «Calmness», «Concerns about delivery and losing control» and «Concern for the child»). The multidimensional structure of the W-DEQ-B is also confirmed by earlier research from different countries [32,33,34,35,36,37], albeit the number of factors differs amongst them. As a result, some studies identified six factors [33,35], four factors [34,36,37], and three factors [32]. The number of items also differs throughout the various tool versions. The W-DEQ-B has 14 items [32], 32 items [33,37], and 33 items [34,35,36] in some versions. Variations in factor structures between studies suggest that FOC may manifest differently depending on culture. Women’s fears may be influenced by cultural perceptions of childbirth as a risky medical procedure. There are differences in the ways that women’s fears appear within their social and cultural environment. Therefore, cultural elements that impact the development of FOC include women’s attitudes toward natural childbirth and their experiences with crowded birthing rooms [35,45,46,47]. Summarizing, the six-factor structure of GrW-DEQ-B is in line with the number of the factor structure of two previous studies [33,35] and is consistent with three earlier studies [34,35,36] regarding the number of items. The GrW-DEQ-B’s multidimensional structure partly reflects the characteristics and substance of Greek women’s postpartum FOC.

The six-factor structure of the GrW-DEQ-B shares the same nomenclature as the GrW-DEQ-A [30], with the exception that the items are not precisely the same. Comparing the factors of the GrW-DEQ-A [30] with those of the GrW-DEQ-B, the following results were obtained: the factor «Lack of feeling lonely» of the GrW-DEQ-B additionally includes items 6, 9, 16 and 23, while it does not include item 31 that was in the corresponding factor of the GrW-DEQ-A; the factor «Lack of self-efficacy» of version B additionally includes items 1 and 21, while it does not include items 6, 9 and 23 that were in the corresponding factor of version A; the factor «Lack of positive anticipation» of the GrW-DEQ-B additionally includes item 31, while it does not include item 21 that was in the corresponding factor of the GrW-DEQ-A; the factor «Calmness» of version B also includes item 2, while it does not include item 16 that was in the corresponding factor of version A; the factor «Concerns about delivery and losing control» does not include items 1 and 2 that were in the corresponding factor in version A; the factor «Concern for the child» is exactly the same. The fact that the two versions of the GrW-DEQ were completed at different times—before and after childbirth—can be attributed to the factors of the two versions that do not contain exactly the same items. Additionally, it is probable that some women will experience and manifest FOC in different ways prior to and following delivery. Also, almost all dimensions of the two versions of the GrW-DEQ (GrW-DEQ-A and GrW-DEQ-B) were significantly correlated with each other.

Since the Cronbach’s α for each of the six factors was greater than 0.7, indicating a reliable scale, this study’s findings suggest that the GrW-DEQ-B has an appropriate internal consistency. Moreover, considering nearly all the GrW-DEQ-B’s factors showed significant positive correlations with one another, the convergent validity findings were considered acceptable. The majority of GrW-DEQ-B’s factors are correlated with the EPDS, a tool used to measure depressive symptomatology, according to the results of the divergent validity analysis. In fact, it was observed that the GrW-DEQ-B and the EPDS had a largely low degree of correlation, indicating that the conceptual substance of the two instruments differs.

According to the results of the present study, the Greek version of the W-DEQ-Β has good psychometric properties. Therefore, the use of the GrW-DEQ-B may be an effective way to screen and measure postpartum FOC in Greek women. In clinical practice, midwifery healthcare professionals need to be able to distinguish between a level of FOC that is considered manageable by women on a daily basis and a level of FOC that requires support beyond routine maternity care [48]. The availability and application of appropriate psychometric instruments that have been validated and proven to be trustworthy are necessary to accomplish this. Furthermore, GrW-DEQ-B’s validation enables it to offer a more comprehensive view of Greek women’s FOC when combined with the GrW-DEQ-A [30]. However, regardless of the above, recognizing GrW-DEQ-B’s acceptable psychometric characteristics is an essential first step in obtaining more objective data and understanding the psychological components of postnatal FOC and its effects on clinical practice.

There are several limitations to the current study that should be noted. First, the majority of the sample was employed, married or cohabiting, from the capital, and had a quite satisfactory educational level. As a result, the results cannot be safely generalized to the whole country’s population. There may be differences in how women’s FOC manifests in rural and urban areas of the same country. As a result, women from rural areas may experience issues, including fewer healthcare providers and reduced health services. The awareness that there might not be sufficient staff available to care for every woman could be one of the factors that contribute to FOC [49]. However, it has also been found that FOC levels in a capital city were higher than those recorded in women in the rural area of the same country. Therefore, a woman’s perception of childbirth and her FOC levels may be influenced by her place of residence [50]. Consequently, more research on the instrument, including participants with a wider range of demographic traits, is needed. Additionally, given that this study’s participants were postpartum women with routine prenatal care following low-risk pregnancies, the findings might not apply to postpartum women with complicated pregnancies and no regular prenatal care. Notwithstanding these limitations, the assessment of the reliability and validity of the Greek version of the W-DEQ-B in postpartum women took place for the first time. This enables healthcare professionals in midwifery settings to employ a suitable instrument to comprehend and identify postpartum FOC in Greece. However, more research on this topic needs to be undertaken, particularly replications of this study in postpartum samples after high-risk pregnancies. Future studies including participants with a greater variety of demographic characteristics could also be taken into consideration.

## 5. Conclusions

The findings of the present study support the reliability and validity of the GrW-DEQ version B among Greek postpartum women as a tool for measuring postnatal FOC after low-risk pregnancies.

## Figures and Tables

**Table 1:** Sample characteristics.

	N (%)
Nationality	
Greek	192 (96.0)
Other	8 (4.0)
Occupation	
Employed	158 (79.0)
Unemployed	28 (14.0)
Household	14 (7.0)
Supportive environment	
Yes, at satisfactory level	187 (93.5)
Minimum support	13 (6.5)
Children from previous pregnancies	
Yes	96 (48.0)
No	104 (52.0)
Description of past childbirth experience	
Very positive	20 (21.3)
Mainly positive	50 (53.2)
Very negative	12 (12.8)
Mainly negative	12 (12.8)
Type of past delivery	
Vaginal delivery	84 (87.5)
Caesarean section	12 (12.5)
Visited a specialist for psychological problems in the past	65 (32.5)
Psychotherapy in the past	45 (22.5)
Stressful event during last year	82 (41.0)
Primigravida	104 (52.0)
Present pregnancy	
Planned	126 (63.0)
Unplanned, but desirable	74 (37.0)
Type of present delivery	
Vaginal delivery	159 (80.0)
Caesarean section	40 (20.0)
Skin-to-skin contact in the first hour after delivery	
Yes	157 (78.5)
No	43 (21.5)
Need for psychological support	
Yes	32 (16.0)
No	168 (84.0)

**Table 2:** Factors loadings from EFA and percentages of variance explained.

Item	Lack of Feeling Lonely	Lack of Self-Efficacy	Lack of Positive Anticipation	Calmness	Concerns About Delivery and Losing Control	Concern for the Child
3	0.70					
6	0.55					
7	0.57					
8	0.58					
9	−0.64					
11	0.50					
15	0.74					
16	−0.47					
20	0.56					
23	−0.71					
1		0.55				
4		0.51				
5		0.59				
10		0.63				
13		0.78				
14		0.73				
17		0.51				
18		0.67				
21		0.50				
22		0.58				
28			0.67			
29			0.79			
30			0.80			
31			−0.70			
2				0.56		
12				0.73		
19				0.49		
24				0.49		
25					−0.70	
26					0.65	
27					−0.66	
32						0.88
33						0.90
% Variance explained	15.5	15.2	8.9	7.9	7.8	5.8

**Table 3:** Participants’ scores on the six factors of the GrW-DEQ-B.

	Minimum Value	Maximum Value	Mean (SD)	Median (IQR)
Lack of feeling lonely	0.30	5.00	3.78 (0.95)	4 (3.3–4.5)
Lack of self-efficacy	0.20	4.60	1.81 (0.98)	1.55 (1–2.5)
Lack of positive anticipation	0.00	5.00	0.81 (1.03)	0.5 (0–1.25)
Calmness	0.00	5.00	2.98 (0.98)	3 (2.25–3.75)
Concerns about delivery and losing control	0.00	5.00	1.4 (0.88)	1.33 (0.67–2)
Concern for the child	0.00	5.00	1.09 (1.31)	0.5 (0–1.5)

**Table 4:** Item–total correlations and Cronbach’s α of the GrW-DEQ-B.

Factor	Item	Corrected Item–Total Correlation	Cronbach’s Alpha if Item Deleted	Cronbach’s Alpha
Lack of feeling lonely	3	0.60	0.88	0.89
	6	0.63	0.88	
	7	0.65	0.88	
	8	0.72	0.87	
	9	0.59	0.88	
	11	0.67	0.88	
	15	0.59	0.88	
	16	0.57	0.89	
	20	0.69	0.88	
	23	0.63	0.88	
Lack of self-efficacy	1	0.65	0.90	0.91
	4	0.66	0.90	
	5	0.69	0.90	
	10	0.67	0.90	
	13	0.78	0.89	
	14	0.74	0.89	
	17	0.69	0.90	
	18	0.67	0.90	
	21	0.42	0.91	
	22	0.71	0.90	
Lack of positive anticipation	28	0.61	0.81	0.83
	29	0.73	0.75	
	30	0.79	0.71	
	31	0.53	0.84	
Calmness	2	0.46	0.43	0.70
	12	0.38	0.68	
	19	0.48	0.41	
	24	0.36	0.70	
Concerns about delivery and losing control	26	0.35	0.64	0.72
	25	0.44	0.60	
	27	0.50	0.51	
Concern for the child	32	0.75	-	0.86
	33	0.75	-	

**Table 5:** Correlations between the six factors of the GrW-DEQ-B.

		Lack of Self-Efficacy	Lack of Positive Anticipation	Calmness	Concerns About Delivery and Losing Control	Concern for the Child
Lack of feeling lonely	r	−0.78	−0.46	0.59	−0.40	−0.15
	*p*	<0.001	<0.001	<0.001	<0.001	0.032
Lack of self-efficacy	r	1.00	0.51	−0.53	0.43	0.15
	*p*		<0.001	<0.001	<0.001	0.034
Lack of positive anticipation	r		1.00	−0.28	0.28	0.22
	*p*			<0.001	<0.001	0.002
Calmness	r			1.00	−0.28	−0.10
	*p*				<0.001	0.162
Concerns about delivery and losing control	r				1.00	0.03
	*p*					0.694

**Table 6:** Correlations between the GrW-DEQ-B’s dimensions and EPDS.

		EPDS
Lack of feeling lonely	r	−0.37
	*p*	<0.001
Lack of self-efficacy	r	0.33
	*p*	<0.001
Lack of positive anticipation	r	0.20
	*p*	0.005
Calmness	r	−0.31
	*p*	<0.001
Concerns about delivery and losing control	r	0.12
	*p*	0.117
Concern for the child	r	0.26
	*p*	<0.001

**Table 7:** Correlations between the GrW-DEQ-A and the GrW-DEQ-B.

		Lack of Feeling Lonely (A)	Lack of Self-Efficacy (A)	Lack of Positive Anticipation (A)	Calmness (A)	Concerns About Delivery and Losing Control (A)	Concern for the Child (A)
Lack of feeling lonely (B)	r	0.52	−0.47	−0.22	0.29	−0.36	−0.20
	*p*	<0.001	<0.001	0.002	<0.001	<0.001	0.004
Lack of self-efficacy (B)	r	−0.40	0.49	0.28	−0.19	0.31	0.13
	*p*	<0.001	<0.001	<0.001	0.009	<0.001	0.068
Lack of positive anticipation (B)	r	−0.38	0.30	0.24	−0.23	0.24	0.11
	*p*	<0.001	<0.001	0.001	0.001	0.001	0.137
Calmness (B)	r	0.36	−0.33	−0.12	0.31	−0.27	−0.18
	*p*	<0.001	<0.001	0.082	<0.001	<0.001	0.011
Concerns about delivery and losing control (B)	r	−0.35	0.22	0.26	−0.07	0.26	0.01
	*p*	<0.001	0.003	0.001	0.388	0.001	0.910
Concern for the child (B)	r	−0.20	0.25	0.08	−0.10	0.17	0.40
	*p*	0.005	<0.001	0.278	0.169	0.017	<0.001

## Data Availability

The datasets analyzed in the current study are available from the corresponding author on reasonable request.

## Supplementary Materials

The following supporting information can be downloaded at: https://www.mdpi.com/article/10.3390/healthcare13080896/s1, Table S1: Dimensions and Items of the GrW-DEQ-B.

## Abbreviations

The following abbreviations are used in this manuscript:

α	Cronbach’s alpha
CFA	confirmatory factor analysis
CFI	comparative fit index
CS	Cesarean section
EFA	exploratory factor analysis
EPDS	Edinburgh Postpartum Depression Scale
FOC	Fear of childbirth
GrW-DEQ-A	Greek version of Wijma Delivery Expectancy/Experience Questionnaire version A
GrW-DEQ-B	Greek version of Wijma Delivery Expectancy/Experience Questionnaire version B
KMO	Kaiser–Meyer–Olkin
PCA	Principal component analysis
r	Spearman’s correlation coefficient
RMSEA	root mean square error of approximation
SD	standard deviation
TLI	Tucker–Lewis index
W-DEQ	Wijma Delivery Expectancy/Experience Questionnaire
W-DEQ-A	Wijma Delivery Expectancy/Experience Questionnaire version A
W-DEQ-B	Wijma Delivery Expectancy/Experience Questionnaire version B
