# Supplementary material for: Validation of the Wijma Delivery Expectancy/Experience Questionnaire (Version B) Among Greek Postpartum Women
Source: Healthcare (Basel). 2025 Apr 14;13(8):896. doi: 10.3390/healthcare13080896 (PMC12026961; doi:10.3390/healthcare13080896)
Supplement: Supplementary file 1 [file healthcare-13-00896-s001.zip › healthcare-3534049-supplementary.pdf]

Table S1. Dimensions and Items of the GrW-DEQ-B

Factor	Item
**Lack of feeling lonely**	3
	6
	7
	8
	9
	11
	15
	16
	20
	23
**Lack of self-efficacy**	1
	4
	5
	10
	13
	14
	17
	18
	21
	22
**Lack of positive anticipation**	28
	29
	30
	31
**Calmness**	2
	12
	19
	24
**Concerns about delivery and losing control**	26
	25
	27
**Concern for the child**	32
	33
